# Supplementary material for: Development and evaluation of loop-mediated isothermal amplification for detection of Yersinia pestis in plague biological samples
Source: PLoS One. 2020 Aug 18;15(8):e0237655. doi: 10.1371/journal.pone.0237655 (PMC7437451; doi:10.1371/journal.pone.0237655)
Supplement: S1 Appendix — (PDF) [file pone.0237655.s001.pdf]

REPOBLIKAN'I MADAGASIKARA

Fitiavana - Tanindrazana - Fandrosoana

MINISTERE DE LA SANTE PUBLIQUE

DIRECTION DES URGENCES ET DE LA LUTTE CONTRE LES MALADIES NEGLIGEES

SERVICE DE LUTTE CONTRE LES MALADIES EPIDEMIQUES ET NEGLIGEES

PROGRAMME NATIONAL DE LUTTE CONTRE LA PESTE

ADY AMIN'NY PESTA

POLITIQUE NATIONALE DE LUTTE CONTRE LA PESTE

EDITION 2012

MSANP/DULMN/SLMEN

Préface

Madagascar figure parmi les orne pays dans le monde où la peste sévit encore de façon endémoépidémique.

Beaucoup d'efforts ont été déployés et de nombreux résultats ont marqué l'évolution de la lutte contre ce fléau depuis la création du programme national de lutte contre la peste au sein du Ministère de la santé et du Planning Familial en 1993.

Cependant, beaucoup reste à faire car ce fléau reste encore un problème de santé publique majeure à Madagascar.

En effet, dans les quarante quatre (44) districts sanitaires foyers dans 13 régions, où cette maladie continue à faire des victimes, les mesures de prévention, de sensibilisation et de surveillance doivent être renforcées. C'est la raison de la réactualisation des stratégies de lutte contre la peste afin que la mise en œuvre des activités soit la plus multisectorielle.

Ainsi, en ce début du 3ème millénaire, lançons le défi pour atteindre les objectifs d'éviter l'apparition de peste pulmonaire et de réduire le taux de létalité à moins de 5%.

L'éradication de la maladie n'est pas pour bientôt, mais il est possible d'éviter sa transmission à l'homme et de contrôler son extension,

Aussi, exhorterai-je tous les agents de santé à faire un pas décisif dans la lutte contre la peste en harmonisant des stratégies adéquates et efficaces afin de prévenir la survenue de cas de peste humaine et de diminuer le nombre de décès par peste.

Enfin, j'adresse tous mes remerciements à tous ceux qui ont contribué à la réalisation de ce document qui servira de guide et de référence pour tous les acteurs œuvrant dans la lutte contre cette maladie.

Le MINISTRE DE LA SANTE PUBLIQUE

Docteur Johanita NDAHIMANANJARA

LISTE DES ABREVIATIONS

BMH : Bureau Municipal d'Hygiène

CSB : Centre de Santé de Base

DRSP : Direction Régionale de la Santé Publique

DULMN : Direction des Urgences et de la Lutte contre les Maladies

E.V : Eugène VERG (dénomination d'une souche de vaccin vivant atténué de Girard et Robic)

FODI : Fiche Officielle de Déclaration Individuelle

I.E.C : Information Education Communication

IPM : Institut Pasteur de Madagascar

LCP : Laboratoire Central Peste

MSANP : Ministère de la Santé Publique

OMS : Organisation Mondiale de la santé

ONG : Organisation Nom Gouvernementale

SLMEN : Service de Lutte contre les Maladies Epidémiques et Négligées

SMY : Streptomycine

SSPD : Service de Santé Publique de District

TDR : Test de Diagnostic Rapide Peste

SOMMAIRE

PREFACE
LISTE DES ABREVIATIONS
INTRODUCTION
CONTEXTE
OBJECTIFS
1 - STRATEGIE DE PREVENTION
1.1. Intensification de la lutte contre les rats
1.2. Renforcement du contrôle de la pullulation des puces libres :
1.3. Renforcement des mesures de la protection de l'homme
1 4 Amélioration de la salubrité de l'environnement
2 - STRATEGIE DE MOBILISATION SOCIALE
3 - STRATEGIE DE SURVEILLANCE
3.1. Intensification de la surveillance des réservoirs
*Surveillance de la mortalité murine
* Surveillance de la densité murine
*Surveillance des déplacements des rats dans les villages
3 2 Intensification de la surveillance des puces
3.3Renforcement de la surveillance de la transmission humaine
*.La surveillance communautaire
*.La surveillance médicale
*Suivi épidémiologique des cas de peste pulmonaire
3.4 Intensification des investigations épidémiologiques, épizootiologiques et environnementales
3.5. Renforcement du Laboratoire Central Peste
4 - STRATEGIE DE PRISE EN CHARGE PRECOCE ET CORRECTE
4.1.Le dépistage précoce de-la maladie
4.2.Le diagnostic clinique de «PRESOMPTION»
4 3 L'utilisation du test diagnostic rapide peste par bandelette»
4 4 La notification de chaque cas à l'échelle nationale et internationale
5 - STRATEGIE DE CONTROLE DE L'EXTENSION DE L'EPIDEMIE
5.1 Intensification de la lutte contre la conservation du bacille
5.2 Renforcement de la lutte contre la circulation du bacille
5.3 Intensification du contrôle de la transmission de la maladie
5.4 Amélioration de la lutte contre la propagation de la maladie
6 - STRATEGIE DE RECHERCHES OPERATIONNELLES ET APPLIQUEES
CONCLUSION
ANNEXE 1 : Attribution des taches dans la lutte contre la peste
ANNEXE II : Cadre législatif concernant la peste
ANNEXE III : Méthodes de piégeage des rongeurs et récolte de puces
ANNEXE IV : Indices pullicidiens
ANNEXE V : Traitement de la peste
ANNEXE VI : Conduite à-tenir devant un cas suspect de peste
ANNEXE Vil : Conduite à tenir en cas de décès par peste

INTRODUCTION

Madagascar s'engage dans la lutte contre la peste et l'inscrit parmi les priorités du Gouvernement décrit dans le Document Stratégique de Réduction de la Pauvreté.
Ainsi, dans le but de protéger la population de la peste, le Ministère de la Santé et du Planning Familial définit la Politique Nationale de Lutte contre la peste dans le cadre de la lutte contre les maladies endémiques par la mise en œuvre sur le plan national d'un programme spécifique axée sur :

- la lutte contre les réservoirs et les vecteurs de la peste ;
- la protection des hommes en évitant la transmission de la peste murine à l'homme.

La réactualisation de la Politique Nationale permettra de renforcer les activités de lutte entreprises et de définir de nouvelles stratégies rigoureuses pour la protection de l'homme contre la peste.

La mise en œuvre de cette politique ne serait effective sans l'engagement des toutes les entités œuvrant dans la lutte contre ce fléau.

CONTEXTE

La Peste, maladie transmissible à déclaration obligatoire, est une maladie des rongeurs due au bacille « Yersinia pestis» et transmise à l'homme de façon accidentelle par piqûre de puces vectrices infectées.

La Peste demeure endémique à Madagascar, depuis son introduction en 1898 jter la ville portuaire de Toamasina, lors de la 3ème pandémie qui a débuté en Chine en 1891. Elle a aussitôt atteint les autres ports de l'Ile (Mahajanga, Antsiranana). Elle a ensuite quitté les côtes pour s'étendre sur les hauts plateaux en 1921 pour y entraîner dans les années 30 de véritables épidémies (3000 à 4000 cas). Grâce aux campagnes de vaccination antipesteuse EV de Girard et Robic, aux efforts de l'assainissement et à la découverte de la Streptomycine et des insecticides, le fléau pesteux fut peu à peu contrôlé vers les années 1940 à 1950 (moins de 50 cas par an). Après une période d'accalmie de quelques décennies, la maladie a connu une recrudescence depuis 1989, où plus de 1 000 cas suspects par an en moyenne étaient déclarés, dont 250 cas Confirmés par la bactériologie. La réapparition depuis quelques années de la Peste dans la capitale est un phénomène inquiétant en raison du risque épidémique élevé dans. cette zone d'importante concentration humaine et à bas niveau d'hygiène. La peste a également résurgi en 1991 sous forme d'importante épidémie dans la ville portuaire de Majunga où elle a fait silence durant 63 ans.

Depuis 1995, surviennent chaque année de sévères épidémies dans 36 Services de Santé et du Planning Familial du Distric (SSPD) des Hautes terres Malgaches. Ces foyers, d'altitude moyenne de 800 m, de degré hygrométrique de 85 à 95% et de température moyenne de 25° C à l'exception de Mahajanga.

Ces foyers forment 2 triangles :

Le triangle principal du centre dont les angles sont formés par le lac Alaotra au Nord, Tsiroanomandidy à l'Ouest etAmbalavao au Sud.

Le triangle mineur du Nord dans le massif de Tsaratanàna

Ces foyers présentent généralement les conditions géographiques, climatiques, socioculturelles et économiques requises pour créer des contextes épidémiologiques très favorables à la persistance, à la transmission et à la propagation de la maladie.
Malgré l'existence de traitement gratuit efficace, des conduites à tenir standardisées simples, une tendance à la baisse de la létalité observée au cours des 5 dernières années, celle-ci reste au-dessus du seuil.

Cette situation est due à :

- à la précarité de l'hygiène liée à la pauvreté favorisant le contact Réservoir-Homme ;
- à l'insécurité obligeant les gens à cohabiter avec les animaux domestiques ;
- au mode de stockage des denrées favorisant l'attraction des rats dans les habitations ;
- au retard de prise en charge des cas par enclavement des zones foyers ;

- à l'ingérence des médecins libéraux et des tradipraticiens dans la prise en charge ;
- à la démotivation des partenaires communautaires ;
- à la non implication des autorités locales dans la lutte ;
- aux us et coutumes comme la veillée mortuaire et l'exhumation favorisant la propagation de la maladie ;
- à l'insuffisance d'expérience sur la prise en charge des cas de peste.

La Peste reste actuellement un problème de santé publique qui nécessite une réorientation de la politique de lutte bien établie. Le Programme National de Lutte contre la Peste existe depuis plusieurs années à Madagascar. Il était mené conjointement par le Ministère de la Santé et l' Institut Pasteur de Madagascar (IPM) jusqu'à 1992.

Depuis Octobre 2004, le Programme National de lutte contre la peste est coordonné par le Service de Lutte contre les Maladies Endémiques, rattaché à la Direction des Urgences et de la Lutte contre les Maladies Transmissibles. Le Ministère de la Santé et du Planning Familial a créé en son sein ce service suivant le.décret N° 2004 - 989 du 19 Octobre 2004.

OBJECTIFS

Dans le but de minimiser l'impact de la maladie pesteuse sur le développement socio-économique du pays, les objectifs du Programme de Lutte sont de :

- Prévenir les épidémies de Peste dans les zones foyers et leur extension dans les zones limitrophes : diminuer de moins de 44 les districts atteints ;
- Diminuer les cas de peste confirmés à un chiffre inférieur ou égal à 100 cas ;
- Diminuer d'ici 5 ans de 14 à 5 le taux de létalité ;
- Eviter l'apparition de peste pulmonaire.

Les grands axes stratégiques pour atteindre ces objectifs sont :

- la prévention de la maladie à travers une lutte solide contre les vecteurs et contre les réservoirs ;
- la mobilisation de la société pour leur engagement et leur participation effective dans toutes les activités de prévention et de lutte ;
- la surveillance épidémiologique des foyers pesteux connus et les zones limitrophes :
- la prise en charge précoce et correcte des cas ;
- le contrôle de l'extension de l'épidémie dans les SDSP foyers et les SDSP limitrophes ;
- les recherches opérationnelles et appliquées.

1 - STRATEGIE DE PREVENTION

La prolifération massive des rats constitue un facteur de risque important à l'éclosion d'une épizootie murine et d'une éventuelle épidémie humaine, car elle entraîne une forte probabilité de contacts 'réservoir -réservoir » et «réservoir - homme».

Ainsi la stratégie de prévention concerne la triade épidémiologique impliquée dans la survenue de la Peste : rats -puces -hommes.

1.1. Intensification de la lutte contre les rats

Des actions appropriées seront entreprises, telles que :

- le contrôle de la densité et de la dynamique de la population murine
- l'utilisation familiale des nasses et autres procédés mécaniques afin de capturer vivant les rats ;
- la promotion des mesures d'assainissement et d'hygiène.

__Remarque__ : la dératisation chimique dans les foyers pesteux sans l'avis des responsables sanitaires est à proscrire.

1.2. Renforcement du contrôle de la pullulation des puces libres

Basée sur :

- la capture des rats à l'état vivant durant une campagne de dératisation pour éviter de rompre la symbiose rat puce et la libération massive de puces libres ;
- l'utilisation des insecticides préconisés par le Programme National dans des circonstances particulières.

1.3. Renforcement des mesures de protection de l'homme

En insistant sur :

- l'observation des mesures d'assainissement et d'hygiène ;
- l'adoption de nouveaux comportements mettant l'homme à l'abri des rats et des puces : promotion de rat - proofing externe et interne ;
- l'interdiction de pratique de feux de brousse.

1.4. Amélioration de la salubrité de l'environnement

1.4.1. En milieu urbain

- par la réglementation des dépôts d'ordures ;
- par le fauchage périodique des alentours des habitations dans un périmètre d'au moins 25 mètres.

1.4.2. En milieu rural

- par le regroupement des déchets ménagers au niveau des hameaux et/ou des fokontany ;
- par installation des fosses à ordures et des latrines au moins à 6 mètres de l'habitation ;
- par le débroussaillage périodique des villages.

2 - STRATEGIE DE MOBILISATION SOCIALE

La participation effective et active de la population et des membres clés de la communauté est nécessaire au développement et au succès des activités du Programme de Lutte contre la Peste.

Ainsi, la stratégie de la mobilisation sociale sera axée sur :

- l'intensification des plaidoyers incluant la mobilisation des ressources est l'engagement des dirigeants et des partenaires à tous les niveaux ;
- l ' élaboration d'une stratégie de communication pour la prévention et la lutte contre la peste en vue d'harmoniser les informations et les messages à transmettre aux populations ;
- l'amélioration des compétences des acteurs en matière de lutte contre la peste ;
- la re dynamisation d'un comité local de santé intégrant toutes les entités locales. Ce comité local de santé joue le rôle de coordonnateur, de promoteur, de contrôleur et de soutien dans la mise en œuvre des actions à réaliser.
- l'intensification de la sensibilisation par l'approche par les pairs et enfant ;
- le développement d'une collaboration franche avec les médecins à profession libérale et les tradipraticiens pour le dépistage précoce des cas suspects de peste.

3 - STRATEGIE DE SURVEILLANCE

Afin de diminuer le risque d'une éclosion épidémique de peste humaine, la surveillance épidémiologique est primordiale, notamment celle de l'épizootie murine qui est la première intervention. En effet, elle constitue la base de la détection précoce de la maladie, de son extension géographique et permet d'évaluer l'importance de l'endémie murine. Cette stratégie de surveillance doit être accès sur la triade épidémiologique : réservoir - puces - homme mais également à la surveillance de l'environnement pour permettre d'entreprendre des mesures de lutte d'urgence afin d'éviter l'apparition d'autres cas humains.

3.1. INTENSIFICATION DE LA SURVEILLANCE DES RESERVOIRS

La surveillance de la mortalité murine entreprise de façon permanente au niveau de la communauté et par la communauté occupe une place importante pour éviter la transmission de la maladie à l'homme et sa propagation. Les épidémies de peste étant toujours précédées d'épizootie murine passée inaperçue. Il est primordial de confirmer qu'une mortalité murine observée est due à la peste. ***La découverte de rat mort exige une surveillance d'un oasis après l'apparition du dernier cas murin.***

Ainsi, l'intensification de la surveillance des réservoirs est basée sur :

- la surveillance de la mortalité murine par la collecte systématique des données relatives :
  - aux sources d'infection humaine,
  - aux facteurs écologiques locaux,
  - aux activités humaines qui peuvent représenter pour l'homme des risques importants d'exposition à la peste,
  - à la tendance de l'épidémiologie et de l'épizootiologie de la peste dans une région donnée ;
- la surveillance de la densité murine par :
  - la détermination des indicateurs de risque,
  - la déclaration obligatoire aux autorités sanitaires compétentes de toute prolifération anormale des rongeurs ;
- la surveillance des déplacements des rats dans les villages par :
  - le repérage des facteurs favorisants : périodes de récolte et des cataclysmes naturels. ;
- la surveillance des autres animaux sensibles à la peste (lapin, cobaye, chien, chat) par examen et analyse de leurs cadavres.

3.2. INTENSIFICATION DE LA SURVEILLANCE DES PUCES

basée sur :

- la détermination de l'indice pullicidien. (Annexe IV)

3.3. RENFORCEMENT DE LA SURVEILLANCE DE LA TRANSMISSION HUMAINE

Ce renforcement sera axée sur :

- la surveillance communautaire par
  - dépistage précoce des cas suspects
  - recherche des décès successifs dans une famille ou dans un village
- la surveillance médicale par
  - identification systématique de la source de l'infection ;
  - détermination de l'étendue de toute activité épizootique ;
  - recherche active d'autres cas humains.
- le suivi épidémiologique des cas de peste pulmonaire par :
  - l'étude de l'évolution des paramètres cliniques pour déterminer la durée d'isolement et la diminution du taux d'attaque.

* La surveillance des décès suspects de peste par investigation de toute cause et application des mesures adéquates pour éviter l'apparition d'épidémie humaine

3.4. INTENSIFICATION DES INVESTIGATIONS EPIDEMIOLOGIQUE, EPIZOOTIQUE ET ENVIRONNEMENTALE

L'intensification sera basée sur :

La détermination de l'étendue de l'épidémie et/ ou de l'épizootie associée au cas humain initial ;
* l'identification des zones de risque potentiel pour l'homme ;
* l'identification des facteu.rs favorisants :
  - types de végétations prédominantes
  - types d'utilisation du sol : agriculture, zone résidentielle, industrielle ou autre,
  - types d'infrastructure : les routes, les chemins de fer, les aéroports et les ports maritimes,
  - types d'habitation présents
  - types d'utilisation des habitations

3,5. RENFORCEMENT DU LABORATOIRE CENTRAL PESTE

Le laboratoire Central de la Peste avec le Service de Lutte contre les Maladies Epidémiques et Négligées (Programme de Lutte contre la Peste), l'Entomologie et l'Epidémiologie de l'IPM (regroupés en une unité Peste), concourent aux activités de laboratoire pour le diagnostic biologique de la peste et de Centre Collaborateur QMS.

Ainsi, ce renforcement consiste :
* à la mise à disposition des moyens et équipements de diagnostic .
* au développement de la compétence des personnels.

4 - STRATEGIE DE PRISE EN CHARGE PRECOCE ET CORRECTE

A Madagascar, le ***traitement*** de la peste est institué ***gratuitement*** comme dans tous les maladies à haut potentiel épidémique.

La précocité, la rapidité et l'effectivité de tous ces éléments de la prise en charge garantissent l'éviction d'une éventuelle éclosion épidémique et constituent un préalable pour réduire la mortalité liée à la peste

Ainsi, cette stratégie sera axée sur quatre points essentiels :

4.1. LE DEPISTAGE PRECOCE DE LA MALADIE

Le **dépistage précoce** de la maladie avec la participation du malade, de la famille, de la population ; à partir des signes suspects (forte fièvre, asthénie, adénopathie douloureuse pour la forme bubonique et fièvre brutale avec toux, douleur thoracique et crachats sanguinolents pour la forme pulmonaire).

Ce « **Diagnostic de suspicion communautaire** » est à intégrer dans le système d'alerte du système de santé et dans le paquet minimum d'activités communautaire.

Ce dépistage précoce de la maladie comporte :
- le renforcement du système d'alerte depuis la famille jusqu'aux structures hiérarchiques de prise en charge ;
- la disponibilité permanente des stocks de sécurité peste (médicaments, insecticides, outils et matériels de prélèvement) ;
- l'amélioration de la compétence des personnels par recyclage et supervisions fonnatives périodiques et régulières des Formations Sanitaires et SDSP foyers de peste.

4.2. LE DIAGNOSTIC CLINIQUE DE «PRESOMPTION»

Le **diagnostic clinique de «présomption»** posé par tout le professionnel de santé public ou privé au vue du contexte clinique et épidéminlogique

L'instauration rapide de la prise en charge du malade, des entourages de l'habitation permet d'éviter l'extension de l'épidémie.

Ainsi, les activités ci-après doivent être instaurées rapidement :
- prélèvement du malade,
- traitement,
- chimioprophylaxie des contacts,
- désinfection et/ou,
- désinsectisation du foyer infecté,
- surveillance du milieu infecté pendant 12 jours après le dernier cas humain.

4.3. L'UTILISATION DU TEST DIAGNOSTIC RAPIDE PESTE PAR BANDELETTE

L'utilisation du test diagnostic rapide peste par bandelette est obligatoire pour confirmer le diagnostic clinique.

4.4. LA NOTIFICATION OBLIGATOIRE DE CHAQUE CAS A L'ECHELLE NATIONALE ET INTERNATIONALE

Elle consiste à enregistrer clairement tous les renseignements nécessaires et utiles concernant chaque malade suspect et le contexte épidémiologique sur le registre quadrifolio ; « fiche officielle de déclaration individuelle» dont un exemplaire sera envoyé avec le prélèvement au Laboratoire Central Peste - Antananarivo - BP 1274 IPM pour la surveillance de la sensibilité des souches aux antibiotiques et pour la déclaration hebdomadaire internationale (DULMN/SLMEN).

5 STRATEGIE DE CONTROLE DE L'EXTENSION DE L'EPIDEMIE

Dans tous les cas, le contrôle de l'extension de la Peste nécessite une intervention rapide à tous les niveau K de lu chaîne épidémiologique. Quatre grandes lignes d'actions sont préconisées :

5.1. INTENSIFICATION DE LA LUTTE CONTRE LA CONSERVATION DU BACILLE

sera axée sur :
- La stérilisation ou la désinfection des pièces ou cases souillées lors du décès d'un malade pesteux ou en présence d'un cas de peste pulmonaire, par utilisation des désinfectants : Crésyl à 5% . Eau de Javel à 10%; HTH à 2%.
- Le traitement par incinération des rats morts suspects ou capturés pour éliminer leurs puces parasites infectés, et les bacilles pesteux ayant colonisé leurs corps ;
- L'utilisation des propriétés bactéricides des ultraviolets solaires sur le sol souillé et les gîtes à rats mis à nus par des débroussaillages larges et périodiques.

5.2. RENFORCEMENT DE LA LUTTE CONTRE LA CIRCULATION DU BACILLE

sera axée sur :

la désinsectisation ciblée et localisée autour d'un cas ou en réalisant une campagne de désinsectisation plus élargie lors d'une épidémie ;
la suppression d'aire vitale pour les rats à proximité de l'habitation de l'homme ;
la réduction du nombre de réservoirs et de vecteurs circulants par application de procédés mécaniques.

5.3. INTENSIFICATION DU CONTROLE DE LA TRANSMISSION DE LA MALADIE

pour éviter le contact homme - réservoir et homme - vecteur par :

- application des méthodes dites « **d'exclusion** » ou « **rat proofing** » ;
- application des mesures d'hygiène individuelle, familiale et de environnementale;
- prise en charge correcte des rats morts.

5.4. AMELIORATION DE LA LUTTE CONTRE LA PROPAGATION DE LA MALADIE

La lutte contre la propagation de la maladie sera améliorée par :

- la prise en charge précoce et correcte des cas humains ;
- le traitement adéquat des corps des rats trouvés morts et des malades suspects ;
- la surveillance de la circulation des personnes suspectes et des colis de denrées alimentaires particulièrement les céréales provenant d'une zone infectée ;
- le respect des règles prescrites en cas de décès suspect ;
- la surveillance du milieu infecté ;
- la suppression des sources de contamination :
- le respect du délai de sept ans entre la date de décès et le moment d'exhumation d'un décès de peste (Famadihana) ;
- la notification obligatoire des cas suspects de peste d'un District à un autre dans un délai de vingt quatre (24) heures ;
- l'interdiction de transfert de la dépouille mortelle avant un délai de sept ans.

6 -STRATEGIE DE RECHERCHES OPERATIONNELLES APPLIQUEES

Les activités de recherche opérationnelle pour la réorientation et l'amélioration des stratégies et des moyens de lutte les plus efficaces et les plus efficients en matière de peste sont à poursuivre :

- recherche comportementale ;
- recherche sur les puces vectrices et les résistances aux insecticides, sur les réservoirs et le cycle épidémiologique de la peste ;
- recherche sur la viabilité du bacille de Yers in dans un cadavre enterré non désinfecté ;
- recherche sur la relation entre l'écologie et la persistance de l'endémie pesteuse.

CONCLUSION

Cette Politique Nationale servira de cadre institutionnel aux responsables de santé publique, aux partenaires et aux autres secteurs œuvrant dans la lutte contre la peste, à améliorer leur contribution dans la réalisation de toutes les activités de lutte dont les mesures d'application techniques sont consignées dans le ***«Guide Technique sur la Peste»***.

C'est également un point de départ fondamental pour la mise en œuvre des principales stratégies envisagées et adaptées aux besoins prioritaires du Pays.

Elle constitue ainsi, le fondement de toute prise de décision et la base nécessaire à l'élaboration des plans annuels de travail à tous les niveaux du système de santé.

L'efficience attendue ainsi que les actions guidées dans ce document resteront vaines tant que les autres secteurs publics ou privés ne se trouvent pas en mesure d'entreprendre des activités convergentes de lutte en vue d'atteindre la *diminution du taux de létalité par peste en moins de 5 et l'absence de peste pulmonaire.*

ANNEXE 1
ATTRIBUTION DES TACHES DANS LA LUTTE CONTRE LA PESTE

Programme National de Lutte contre la Peste

1 - Former les personnels des centres de soins sur :
- les connaissances de base de l'épidémiologie et de la clinique de la peste
- la technique de surveillance épidémiologique de la peste,
- les gestes techniques à visée diagnostique,
- la conduite du traitement,
- l'intervention autour d'un cas suspect,
- l'exécution d'une éducation sanitaire ciblée à la population.

2 - Approvisionner les centres de soins en :
- supports didactiques,
- médicaments spécifiques,
- matériels nécessaires aux prélèvements biologiques à visée diagnostique,
- insecticides et désinfectants pour intervention ciblée,
- matériels d'épandage,
- formulaires de déclaration obligatoire des cas suspects de peste,

3 - Superviser la bonne exécution des méthodes de lutte contre la peste au niveau des centres de soins.
4 - Effectuer les interventions lors des épidémies dépassant les capacités des structures périphériques
5 - Mettre à jour de façon régulière les consignes relatives à la lutte contre la peste et aux conduites à tenir en matière de prise en charge des cas peste ;
6 - Informer le public sur le plan national. ;
7 - Informatiser et analyser les données épidémiologiques. ;
8 - Evaluer les activités de lutte contre la peste

Rôle du Laboratoire Central de la Peste MSANP/IPM

Ils œuvrent différentes activités capitales dans le cadre du programme national :
1- Réceptionner tous les prélèvements provenant des informations sanitaires périphériques.
2 - Tenir et mettre à jour le fichier central informatisé des cas déclarés associant les données bactériologiques et épidémiologiques.
3 - Assurer le diagnostic de confirmation en bactériologie, en sériologie et en Test de Diagnostic Rapide peste (TDR).
4 - Assurer la surveillance de la sensibilité de *Yersinia pestis* aux antibiotiques.
5 - Tenir le soucher de *Y. pestis.*
6 - Transmettre de façon hebdomadaire les résultats de l'examen de laboratoire cas déclarés et confirmés à la DULMN/SLMEN/ Programme National de Lutte contre la Peste.
7 - Participer à la surveillance de la peste murine avec les entités concernées (Programme de Lutte contre la Peste, la DRSP, le SDSP et le BMH).
8 - Former et superviser les personnels des Formations sanitaires périphériques sur l'utilisation du TDR
9 - Produire le TDR, les kits de prélèvement et de transport.

Le maintien du rôle du LCP est primordial pour le bon fonctionnement du programme national. Dans ce cadre, il est indispensable que ce dernier dispose des ressources adéquates (humaines, matérielles et financières).

Complémentairement aux activités de santé publique, l'Unité peste joue un rôle fondamental en terme de recherches appliquées telle le développement d'outils de diagnostic et de survol lance, le développement de nouveaux vaccins et études immunologiques, des investigations épidémiologiques, entomologiques et rodontologiques selon les besoins et les demandes du Ministère de la Santé Publique. Les activités de recherche sont indispensables pour les éventuelles réorientations des stratégies du programme.

Bureau Municipal d'Hygiène (BMH)

1 - Mettre en œuvre les activités de riposte autour des cas et les mesures de prévention contre la peste
2 - Mettre en route la chimioprophylaxie des contacts.
3 - Réaliser la désinsectisation et la désinfection en cas de peste pulmonaire, en cas de décès suspect des foyers infectés.
4 - Surveiller le milieu infecté pendant 12 jours après la détection des cas suspects.
5 - Enregistrer et contrôler les décès suspects.
6 - Contrôler et suivre la mise en bière, inhumation des morts suspects de peste.
7 - Informer les entités oeuvrant dans la lutte.
8 - Participer à la surveillance de la peste murine avec les entités concernées (Programme de Lutte contre la Peste, la DRSP, le SDSP et le BMH).

Direction Régionale de la Santé Publique (DRSP)

1 - Coordonner les activités techniques et administratives au niveau régional
2 - Superviser les districts sanitaires foyers de peste.
3 - Apporter l'appui technique et logistique en cas de besoin SDSP foyers pesteux,
4 - Appuyer les districts sanitaires et les centres de santé périphériques en cas d'épidémie de peste.
5 - Evaluer les activités de lutte contre la Peste dans la Région.
6 - Participer à la surveillance de la peste murine avec les entités concernées : le SLMEN (Programme National de Lutte contre la Peste), la DRSP, le SDSP et le BMH.

Service de District de la Santé Publique (SDSP)

1 - Eriger ou redynamiser les comités locaux de surveillance peste.
2 - Former tous les intervenants en matière de lutte contre la peste.
3 - Apporter l'appui technique et logistique en cas de besoin aux CSB foyers pesteux.
4 - Assurer le stock d'urgence peste dans les CSB.
5 - Collaborer avec les partenaires potentiels (Autorités administratives locales et Sécurité publique).
6 - Etablir la carte épidémiologique peste.
7 - Enregistrer et définir les cas suspects de peste.
8 - Remplir les formulaires de notification (FODI) à envoyer dans un delai maximum de 10 jours.
9 - Envoyer la fiche d'alerte au Service de Surveillance Épidémiologique et les prélèvements au Laboratoire Central IPM.
10 - Envoyer les rapports d'activités au SLMEN/DULMN.
11 - Appuyer les Centres de Santé périphériques en cas d'épidémie de peste.
12 - Assurer la supervision formative des CSB à problèmes.
13 - Participer à la surveillance de la peste murine avec les entités concernées: SLMEN, Programme National de Lutte contre la Peste, , la DRSP, le SDSP et le BMH.
14 - Commander les besoins en Kit de prélèvement et TDR
15 - Entreprendre une investigation devant deux décès successifs dans un délai de six jours dans une localité.
16 - Contrôler et suivre la mise en bière, inhumation des morts suspects de peste.

Centre de Santé de Base (CSB)

1. - Eriger ou redynamiser les comités locaux de surveillance peste,
2. - Sensibiliser la population sur les mesures de prévention et sur les mesures de riposte contre la peste.
3 -Assurer la prise en charge des cas suspects :
   * poser le diagnostic de peste ;
   * faire le prélèvement ;
   * traiter le malade ;
   * donner la chimioprophylaxie au contact proche ;
   * faire la désinsectisation du foyer infecté ;
   * surveiller le milieu pendant 12 jours :
     - dépister les nouveaux cas de maladie ;
     - surveiller l'évolution des contacts vers le stade maladie ;
     - surveiller la présence éventuelle des rats morts :
     - surveiller la pullulation des puces libres après une opération de désinsectisation ;
     - effectuer la mise en bière en cas de décès par peste.
4 - Effectuer les prélèvements à visée diagnostique.
5 - Notifier les cas suspects aux hiérarchies sanitaires.
6 - Effectuer l'intervention épidémiologique autour des cas suspects.
7 - Diffuser les informations sanitaires à la population desservie par la Formation Sanitaire.
8 - Etablir la carte épidémiologique peste.
9 - Envoyer les rapports d'activités à la hiérarchie concernée.
10 - Participer à la surveillance de la peste murine avec les entités concernées : le SLMEN (Programme de Lutte contre la Peste), la DRSP et le BMH.
11 - Collaborer avec les autres prestataires de soins (tradipraticiens, dépositaires de médicaments, Mpiandry, Matrone, Médecin en exercice libéral,...).
12 - Contrôler et suivre la mise en bière, inhumation des morts suspects de peste.

Institut National de Santé Publique et Communautaire (INSPC)

1 - Réactualiser périodiquement l'enseignement sur la peste prodigué aux étudiants en médecine.
2 - Mener des recherches en vue d'améliorer la connaissance sur l'épidémiologie de la peste.

Institut Pasteur de Madagascar (IPM)

1 - Conseiller et appuyer techniquement le Laboratoire Central.
2 - Réaliser des activités de recherche appliquées et opérationnelles.

Collectivités locales/Communales/ONG

1 - Participer au renforcement de l'hygiène et de la salubrité du milieu.
2 - Contribuer au système d'alerte et de riposte.
3- Contribuer à la surveillance épidémiologique.

ANNEXE II

CADRE LEGISLATIF CONCERNANT LA PESTE

1 - Règlement Sanitaire International (1969), troisième édition annulée, OMS Genève 1983, titre V, Chapitre I, article 50 à 60.

2 - Ordonnance n° 62-072 du 29 Septembre 1962 JORM du 12 Octobre 1962, p.2.239 à 2.248, livre IV , titre V Lutte contre la peste, articles 85 à 100) ; portant codification des textes législatifs concernant la Santé Publique.

3 -Arrêté n° 230-SAN du 30 Janvier 1961 (JORM du 04 Février 1961. page 222) ; fixant les modalités du rétablissement des prélèvements post mortem dans certains districts et cantons de la Province de Tananarive.

4 - Note de service n° 1.573 SAN/SG/DSSM/SMS/Sl.MP DU 28 Janvier 1981 relative à la surveillance et au contrôle de l'endémie pesteuse.

5 - Lettre n° 136-SAN/SG/DLMT/PNI.PBC du 9 Janvier 1997 du Directeur de la Lutte contre les Maladies Transmissibles relative au circuit d'information en cas d'éclosion d'épidémie de peste.

6 - Lettre n° 33-SAN/SG/DLMT/PN1.PBC du 18 Juin 1997 du Ministère de la Santé pour . la surveillance de l'éclosion de la maladie et pour le contrôle de la propagation de la maladie,

7 - Décret N° 2004 - 989 du 19 Octobre 2004, rattachant le Service de Lutte contre les Maladies Endémiques à la Direction des Urgences et de Lutte contre les Maladies Transmissibles.

ANNEXE III

METHODES DE PIEGEAGE DES RONGEURS ET RECOLTE DES PUCES

1°) OBSERVATION DES COLONIES DE RONGEURS ET DES SIGNES D'ACTIVITES DES RONGEURS

- Etablir une carte et contrôler la zone périodiquement pour observer les signes visibles d'activité parmi les rongeurs sensibles à la peste

Si ces animaux sont normalement visibles en période calme, leur disparition à la suite d'une épizootie de peste est hautement significative.
Il faut enregistrer le nombre d'animaux observés dans chaque site pendant un laps de temps déterminé.

Si l'on suspecte qu'une épizootie de peste est survenue récemment ou est en cours dans une de ces colonies, il faut inspecter la zone à la recherche de cadavres d'animaux.
Autres signes indicateurs d'une hécatombe de rongeurs sont notamment :

* La présence de mouches nécrophages à l'entrée des terriers ;
* Une mauvaise odeur près des terriers et des terriers mal entretenus ;
* Des puces potentiellement infectées peuvent aussi être récoltées sur les cadavres d'animaux ou dans les terriers abandonnés.

2°) PIEGEAGE DES RONGEURS

Il est important de piéger et d'examiner les rongeurs de manière systématique pour déterminer :

* Les hôtes potentiels de la peste dans une zone donnée ;
* Le nombre et les types de puces qui infestent ces animaux ;
* Si de nouvelles espèces de rongeurs sont entrées dans une zone ;
* Si la quantité d'espèces de rongeurs indigènes a changé de manière significative depuis la dernière période de piégeage ;
* Les densités de population relatives ou absolues ;
* Les structures par âge et le statut reproductif des populations des rongeurs ;
* Les préférences des rongeurs en matière d'habitat ;
* La répartition locale.

% de succès de piégeage = (Nombre de rongeurs/ Nombre de période de piégeage/ Nombre de pièges installés par période) X 100

3°) RECOLTE DES PUCES DANS LES TERRIERS

Les puces peuvent être récoltées dans les terriers de rongeurs par nettoyage ou curetage
Lorsque les puces sont périodiquement échantillonnées par cette méthode, on note souvent que les indices des terriers sont peu élevés entre les épizooties, mais qu'ils augmentent de manière significative lorsque les épizooties provoquent une létalité élevée chez les hôtes.

L'écouvillon à terrier typique est formé d'un câble d'acier ou d'un tuyau de caoutchou dur avec un morceau de coton fixé à son extrémité. Le câble est utilisé pour pousser le tissu à l'intérieur de l'entrée du terrier; les puces le prennent pour leur hôte normal et s'y accrochent. On extrait alors le tissu du terrier et l'on inspecte à la recherche de puces ou on place dans un sac en plastique jusqu'à ce qu'il soit examiné. On peut tuer les pucces dans un sac en plastique par congélation, par anesthésie ou à l'aide d'insecticides.

ANNEXE IV

INDICES PULLICIDIENS

Une méthode d'échantillonnage séquentiel pour déterminer le nombre d'animaux hôtes à échantillonner afin d'obtenir un indice pulicidien fiable pour une relation donnée hôte/puce a été décrite par SCHWAN,
On a trouvé que 20 seulement étaient nécessaires pour établir un indice pullicidien spécifique fiable.

La procédure standard pour la préparation des puces en vue du l'inuculalion est le broyage de mélange de puces (jusqu'à 25 par groupe) à l'aide d'un mortier et d'un pilon et la mise en suspension du broyat dans 2 ml de sérum physiologique

CALCUL D'INDEX

Indice pullicidien spécifique = <u>Nombre de puces de l'espèces A récolté dans l'espèce d'hôte Y</u>
Nombre d'individus de l'espèce hôte Y examiné

Indice pullicidien total = <u>Nombre de puces récoltées (peu importe l'espèce)</u>
Nombre total d'hôte Y examiné

Pourcentage d'hôtes infectés = <u>Nombre de puces de l'espèce Y infestée par l'espèce de puce A x100</u>
Nombre total d'hôte Y examiné

Indice de terrier = <u>Nombre de puces de l'espèce A récoltées dans le terrier de l'espèce hôte Y</u>
(ou nid de terrier) Nombre total de terriers de l'espèce hôte examiné

ANNEXE V

TRAITEMENT DE LA PESTE

1°) Traitement standard spécifique préconisé par le Programme National

1.1. En cas de Peste bubonique :
<u>Médicaments</u> : Streptomycine (SMY) et Sulfaméthoxazole -Triméthoprime
<u>Durée:</u> 8 jours
<u>Schéma</u> :
a) <u>Chez l'adulte:</u>

* SMY : à la dose de 3g par jour en doses fractionnées de 0,5 g les 2 premiers jours et 2 g par jour en doses fractionnées de 1 g le 3ème et le 4ème jour
* Sulfamides: Sulfaméthoxazole - Triméthoprime comprimés à 400/80 mg
Durée : 6 jours à partir du 3è jour du traitement
Doses : 40/8 mg/Kg/jour d'où 6 comprimés par jour jusqu'à 8 à 10 jours

b) <u>Chez l'enfant et l'adolescent, on institue :</u>

* SMY : à la dose de 7.5 mg/Kg/injection toutes les 8 heures du 1er au 5ème jour * relayer par le Sulfaméthoxazole Triméthoprime, à la dose de 40/8mg/Kg/jour à partir du 3ème 'jusqu'au 8ème jour

En pratique, les schémas prescrits par le tableau suivant sont adoptés :

. Age.	Médicaments/forme	1er jour	2ème Jour	3ème jour	4ème jour	5ème jour	6ème jour	7ème jour	8ème jour
Adulte	SMY 1g injectable	0,5 g toutes les 4 heures	0,5 g toutes les 4 heures	1 g matin 1g soir	1 g matin 1 g soir				
	Sulfaméthoxaîolê Triméthoprime cp à 400/80 mg			3 cp matin 3 cp soir	3 cp matin 3 cp soir	3 cp matin 3 cp soir	3 cp matin 3 cp soir	3 cp matin 3 cp soir	3 cp matin 3 cp soir
Plus de 6-15 ans	SMY 1 g injectable	0,5 g toutes les 8 heures	0,5 g toutes les 8 heures	0,5 g toutes les 8 heures	0,5 g toutes les 8 heures	0,5 g toutes les 8 heures			
	Sulfaméthoxazole Triméthoprime cp à 400/80 mg			2 cp matin 2 cp soir	2 cp matin 2 cp soir	2 cp matin 2 cp soir	2 cp matin 2 cp soir	2 cp matin 2 cp soir	2 cp matin 2 cp soir
Plus de 3 - 6 ans	SMY 1 mg Injectable	1/3 fl toutes les 8 heures	1/3 fl toutes les 8 heures	1/3 fl toutes les 8 heures	1/3 fl toutes les 8 heures	1/3 fl toutes les 8 heures			
	Sulfaméthoxazole Triméthoprime cp à 400/80 mg			½ cp matin ½ cp soir	½ cp matin ½ cp soir	½ cp matin ½ cp soir	½ cp matin ½ cp soir	½ cp matin ½ cp soir	½ cp matin ½ cp soir
0-3 ans	SMY 1 g injectable	1/5 fl toutes les 8 heures	1/5 fl toutes les 8 heures	1/5 fl toutes les 8 heures	1/5 fl toutes les 8 heures	1/5 fl toutes les 8 heures			
	Sulfaméthoxazole Triméthoprime cp à 400/80 mg			¼ cp matin ¼ cp soir	¼ cp matin ¼ cp soir	¼ cp matin ¼ cp soir	¼ cp matin ¼ cp soir	¼ cp matin ¼ cp soir	¼ cp matin ¼ cp soir

1.2. En cas de Peste pulmonaire :

<u>Médicament</u> : SMY uniquement
<u>Durée</u> : 8 à 10 jours
<u>Schéma</u> :

Chez l'adulte : 1er et 2ème jour : 4 g en doses fractionnées de 0,5 g toutes les 3 heures
3ème et 4ème jour : 3 g en doses fractionnées de 0,5 g toutes les 4 heures
5ème au 8ème jour : 2 g à raison de 1 g le matin et 1 g le soir

Chez l'enfant et l'adolescent, on préconise une dose de
pendant les 4 premiers jours :7,5 mg/Kg/injection toutes les 3 heures pendant 4 premiers jours
puis 15 mg/kg/injection matin et .soir du 5ème au 8 ème jour.

AGE	Médicaments	1er jour	2ème jour	3ème jour	4ème jour	5ème jour	6ème jour	7ème jour	8ème jour
Adulte	SMY injectable (1g – 1flc) Dose selon Les poids	0,5g toutes Les 3 heures UUUU UUUU	0,5g toutes Les 3 heures UUUU UUUU	0,5g toutes Les 3 heures UUU UUU	0,5g toutes Les 3 heures UUU UUU	1 flc matin 1flc soir UU UU	1 flc matin 1flc soir UU UU	1 flc matin 1flc soir UU UU	1 flc matin 1flc soir UU UU
	SMY injectable	7,5mg/Kg	7,5mg/Kg	7,5mg/Kg	7,5mg/Kg	15mg/Kg	15mg/Kg	15mg/Kg	15mg/Kg
Moins 15 ans	(1g – 1flc) Dose selon Les poids	A chaque Injection UUUU UUUU	A chaque Injection UUUU UUUU	A chaque Injection UUUU UUUU	A chaque Injection UUUU UUUU	A chaque Injection U U	A chaque Injection U U	A chaque Injection U U	A chaque Injection U U

U: nombre d'injection

2°)-. Traitement préconisé par l'OMS en cas d'allergie à la streptomycine
Le chloramphénicol esl aussi, très actif sur *Yersinia pestis*, habituellement réservé aux méningites pesteuses. Une dose de charge de 25 mg/ Kg suivie par un traitement intraveineux de dix jours à la dose de 50 à 75 mg/ Kg/ jour est recommandée par l'OMS (BUTLER). La dose de chloramphénicol étant de 20 à 25 g.

ANNEXE VI

CONDUITE A TENIR DEVANT UN CAS SUSPECT DE PESTE

1°) Les prélèvements biologiques

Le prélèvement de « bubon» pour la forme bubonique et le prélèvement de **«crachat»** pour la forme pulmonaire à pratiquer avant la mise en route du traitement, pour le diagnostic biologique rapide et isolement et à envoyer avec les précautions d'usage, par la voie le plus rapide au Laboratoire Central peste de la Direction des Urgences et de la Lutte contre les Maladies Négligées (DULMN) à l'IPM.

2°) Le traitement précoce des malades

La streptomycine reste l'antibiotique le plus efficace du traitement de la peste (MEYER). Les guérisons atteignent 96% des cas de peste bubonique et 88% dans la peste pulmonaire. Les décès sont dus le plus souvent à un traitement tardif, après la 40ème heure en cas de peste pulmonaire. Le risque de lyse bactérienne massive (POLAND) en cas d'utilisation de dose élevée de strepto-mycine jusqu'à 10 g par jour. Ceci a fait recommander les doses moindres

Le Ministère de la Santé Publique (SLMEN/DULMN) préconise un schéma standard (Annexe V). L'utilisation de la gentamicine est en cours d'étude avec le CDC ATLANTA.

3°) L'identification et la chimioprophylaxie des contacts

On entend par « contact », tout sujet vivant au foyer du malade et toutes personnes ayant été trouvées à proximité du (des) malades suspects 48 heures avant l'apparition des signes ou contact avec un rat mort suspect ou décès suspect.

La chimioprophylaxie des contacts doit être obligatoirement instituée dans un délai inférieur ou égal à deux jours après le dépistage du malade suspect,

3.1. Le médicament préconisé pour la chimioprophylaxie des contacts est la Sulfadoxine (FANASIL 500 ®) ***<u>pour la peste bubonique</u>***, administrée en dose unique :
La sulfadoxine qui est un sulfamide retard protège pendant une période de 15 jours
Chez l'adulte : 4 comprimés
Chez l'enfant et l'adolescent :

- 1 cp pour l'enfant moins de 3 ans
- 1 cp et demi pour l'enfant âgé entre 3 et 6 ans
- 2 cp pour l'enfam plus de 6 ans jusqu'à 12 ans
- 3 cp pour l'adolescent plus de 12 ans jusqu'à 15 ans.

Le Sulfaméthoxazole - Triméthoprime est administré à la dose de 40/8mg/Kg/j pendant 5 jours , à préconiser pour ***<u>la peste pulmonaire.</u>***

4°) La désinsectisation « ciblée et localisée » du foyer infecté

La désinsectisation << ciblée ou localisée » par épandage d'insecticides sous forme de poudre à poudrer (Pyréthrinoïde ou Carbamate) dans la case infectée ainsi que les cases avoisinantes dans un rayon de ***200 mètres***, est une mesure à ne pas omettre, couplée avec la sensibilisation de la population sur les mesures de riposte pour éviter la propagation de l'épidémie. Les cases où l'on a trouvé des rats morts suspects devront être désinsectisées. La rémanence (durée d'efficacité) de ces produits est en moyenne de trois mois.

5°) Surveillance du milieu pendant 12 jours après le dernier cas humain et un mois après le dernier cas murin.

- dépister les nouveaux cas de maladie ;
- surveiller l'évolution des contacts vers le stade maladie ;
- surveiller la présence éventuelle des rats morts ;
- surveiller la pullulation des puces libres après une opération de désinsectisation :
- effectuer la mise en bière en cas de décès par peste.

ANNEXE VII

CONDUITE A TENIR EN CAS DE DECES PAR PESTE

1°) Prise en charge du décès

- Alerter le plus vite possible le responsable de santé le plus proche
- Eviter toutes les visites en attendant l'arrivée de l'agent de santé
- Limiter à six personnes les manipulations du cadavre
- Eviter les veillées mortuaires
- Désinfecter rapidement le cadavre avec chlorure de chaux ou HTH 2%
- Faire la mise en bière
    * cercueil à double paroi (en zinc et en bois)
    * mettre du charbon et chlorure de chaux dans le cercueil en zinc
    * déposer le cadavre à l'intérieur
    * sceller le cercueil

- Enterrement du cadavre hors du caveau familial dans un délai inférieur à 6 heures après l'heure du décès
- Interdiction de transport de la dépouille mortelle hors du lieu de décès

2°) Protection de l'entourage

- Dépistage actif des cas « maladie » chez l'entourage
- Chimioprophylaxie des contacts à toutes personnes en contact avec le décès
- Désinsectisation et désinfection du foyer infecté
- Chimioprophylaxie des personnes qui ont manipulé le cadavre
- Surveillance de l'entourage et des personnes qui ont manipulé le cadavre pendant 12 jours
- Surveillance du milieu pendant 12 jours
