# Supplementary figures and images for: Development and evaluation of loop-mediated isothermal amplification for detection of Yersinia pestis in plague biological samples
Source: PLoS One. 2020 Aug 18;15(8):e0237655. doi: 10.1371/journal.pone.0237655 (PMC7437451; doi:10.1371/journal.pone.0237655)

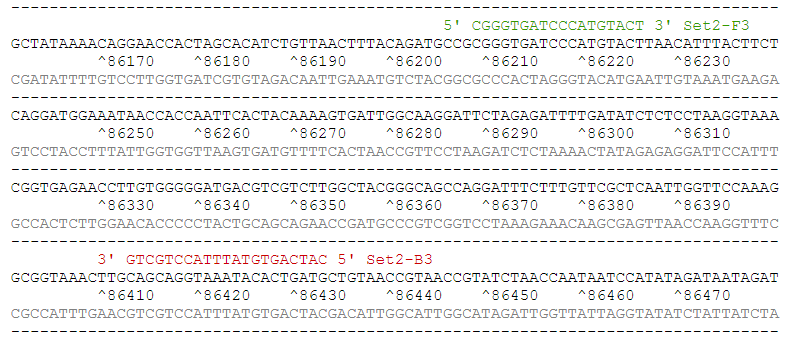

Supplement: S2 Appendix — (TIF) [file pone.0237655.s002.tif]
