# Supplementary material for: Development and evaluation of loop-mediated isothermal amplification for detection of Yersinia pestis in plague biological samples
Source: PLoS One. 2020 Aug 18;15(8):e0237655. doi: 10.1371/journal.pone.0237655 (PMC7437451; doi:10.1371/journal.pone.0237655)
Supplement: S1 Raw images — (PDF) [file pone.0237655.s003.pdf]

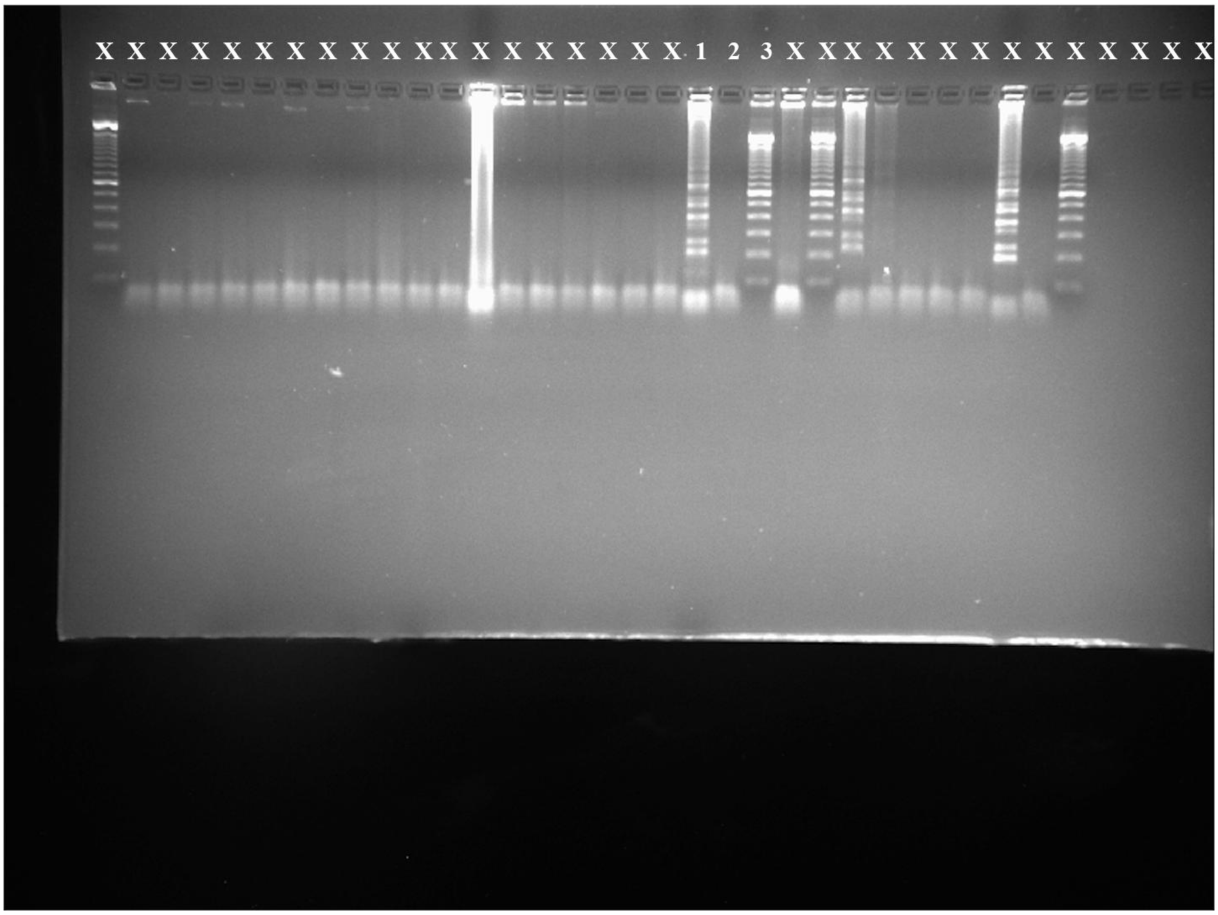

Original gel image supporting Fig 1 (B). Electrophoresis of LAMP amplicons on 1.5 % agarose gel stained with ethidium bromide. Gel Doc XR+ System (Bio Rad) with Quantity One 1-D Analysis Software were used to capture the image. Lane 1, *Yersinia pestis* (59/15); Lane 2, No DNA template; Lane 3, 100 bp DNA ladder; 33 Lanes X in order, 100 bp DNA ladder, *Yersinia enterocolitica*, extraction control, *Yersinia pseudotuberculosis*, *Enterobacter cloacae*, *Escherichia coli*, *Shigella sonnei, Proteus mirabilis*, *Serratia odorifera*, *Serratia marescens*, *Pseudomonas aeruginosa*, *Staphylococcus aureus*, *Mycobacterium tuberculosis* (1), *M. tuberculosis* (2), *M. tuberculosis* (3), *M. tuberculosis* (4), *Plasmodium vivax*, *Plasmodium falciparum*, *Taenia solium, E. coli* 2nd test, 100 bp DNA ladder, *Y. pestis* (diluted 10^{-1}), *Y. pestis* (diluted 10^{-2}), *Y. pestis* (diluted 10^{-3}), *Y. pestis* (diluted 10^{-4}), *Y. pestis* (diluted 10^{-5}), *Y. pestis* non diluted, No DNA template, 100 bp DNA ladder, empty lane, empty lane, empty lane, empty lane.

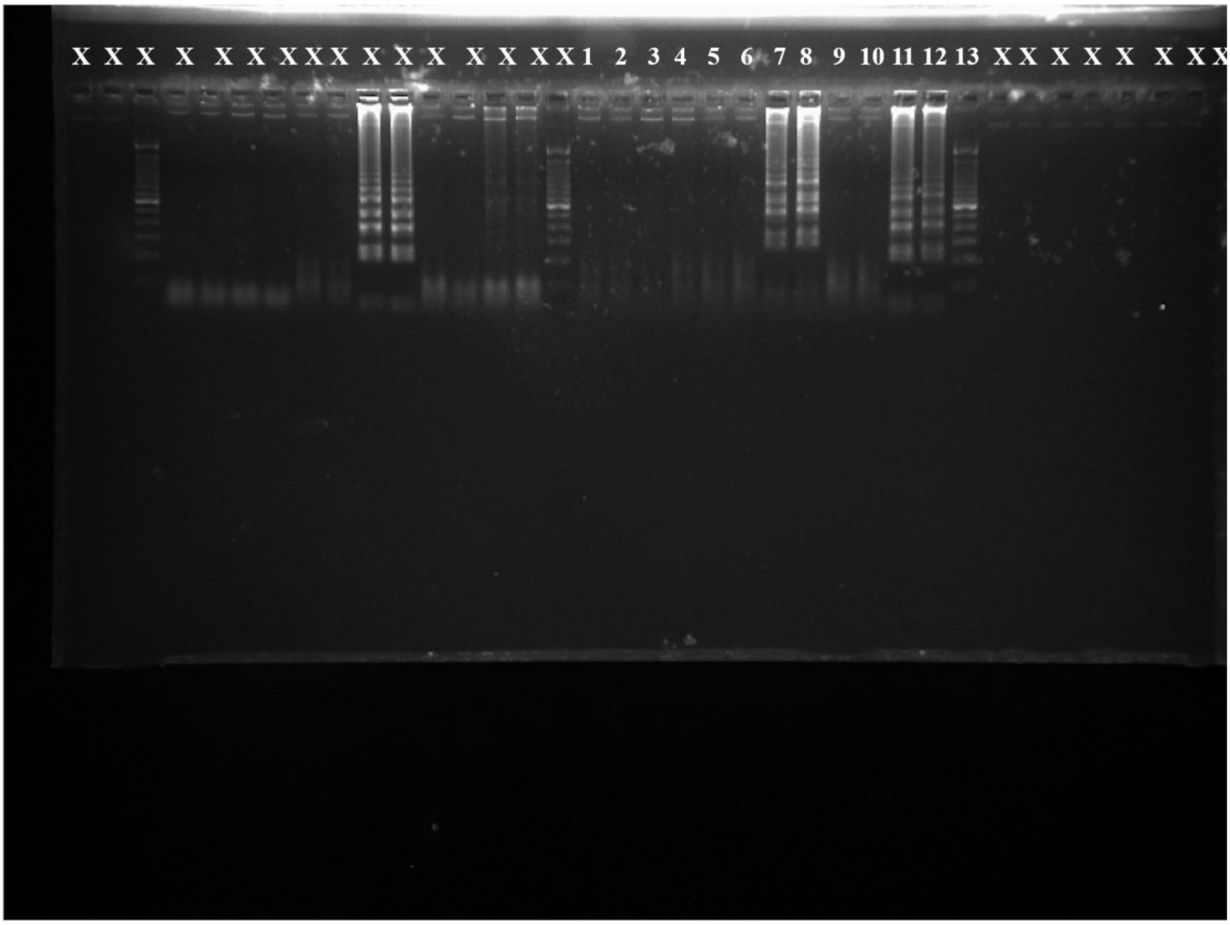

Original gel image supporting Fig 2 (A). Electrophoresis of LAMP amplicons on 1.5 % agarose gel stained with ethidium bromide. Gel Doc XR+ System (Bio Rad) with Quantity One 1-D Analysis Software were used to capture the image. Lane 1, no DNA template; Lane 2, no DNA template, Lane 3, *Yersinia pestis* (59/15); Lane 4, *Y. pestis* (59/15); Lane 5, no DNA template; Lane 6, no DNA template, Lane 7, *Y. pestis* (59/15); Lane 8, *Y. pestis* (59/15); Lane 9, no DNA template; Lane 10, no DNA template, Lane 11, *Y. pestis* (59/15); Lane 12, *Y. pestis* (59/15); Lane 13, 100 bp DNA ladder; 24 Lanes X in order, empty lane, empty lane, 100 bp DNA ladder, no DNA template, no DNA template, *Y. pestis* (59/15), *Y. pestis* (59/15), no DNA template, no DNA template, *Y. pestis* (59/15), *Y. pestis* (59/15), no DNA template, no DNA template, *Y. pestis* (59/15), *Y. pestis* (59/15), 100 bp DNA ladder, empty lane, empty lane, empty lane, empty lane, empty lane, empty lane, empty lane, empty lane.

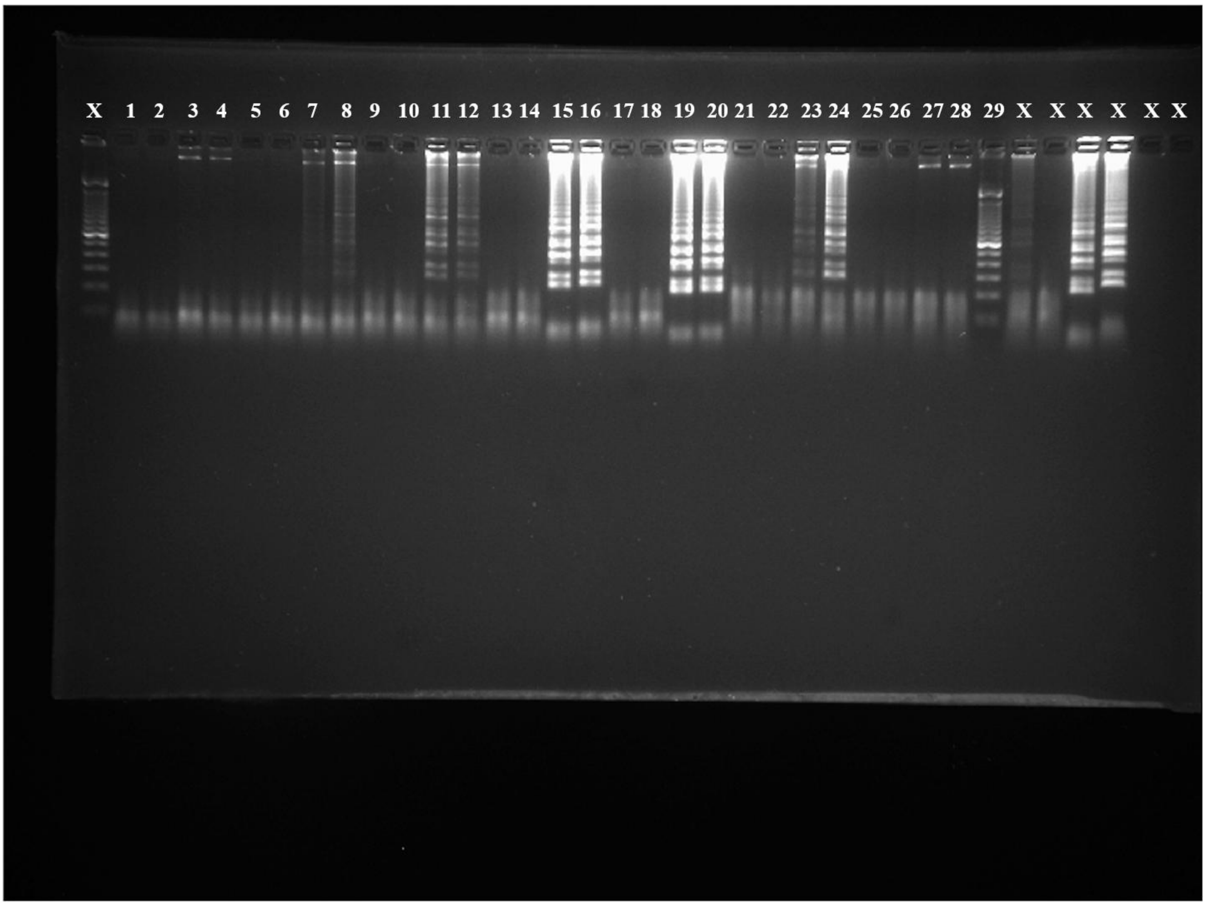

Original gel image supporting Fig 2 (B). Electrophoresis of LAMP amplicons on 1.5 % agarose gel stained with ethidium bromide. Gel Doc XR+ System (Bio Rad) with Quantity One 1-D Analysis Software were used to capture the image. Lane 1, no DNA template; Lane 2, no DNA template; Lane 3, *Yersinia pestis* (59/15); Lane 4, *Y. pestis* (59/15); Lane 5, no DNA template; Lane 6, no DNA template; Lane 7, *Y. pestis* (59/15); Lane 8, *Y. pestis* (59/15); Lane 9, no DNA template; Lane 10, no DNA template; Lane 11, *Y. pestis* (59/15); Lane 12, *Y. pestis* (59/15); Lane 13, no DNA template; Lane 14, no DNA template; Lane 15, *Y. pestis* (59/15); Lane 16, *Y. pestis* (59/15); Lane 17, no DNA template; Lane 18, no DNA template; Lane 19, *Y. pestis* (59/15); Lane 20, *Y. pestis* (59/15); Lane 21, no DNA template; Lane 22, no DNA template; Lane 23, *Y. pestis* (59/15); Lane 24, *Y. pestis* (59/15); Lane 25, no DNA template; Lane 26, no DNA template; Lane 27, *Y. pestis* (59/15); Lane 28, *Y. pestis* (59/15); Lane 29, 100 bp DNA ladder; 7 Lanes X in order 100 bp DNA ladder, no DNA template, no DNA template, *Y. pestis* (59/15), *Y. pestis* (59/15), empty lane, empty lane.

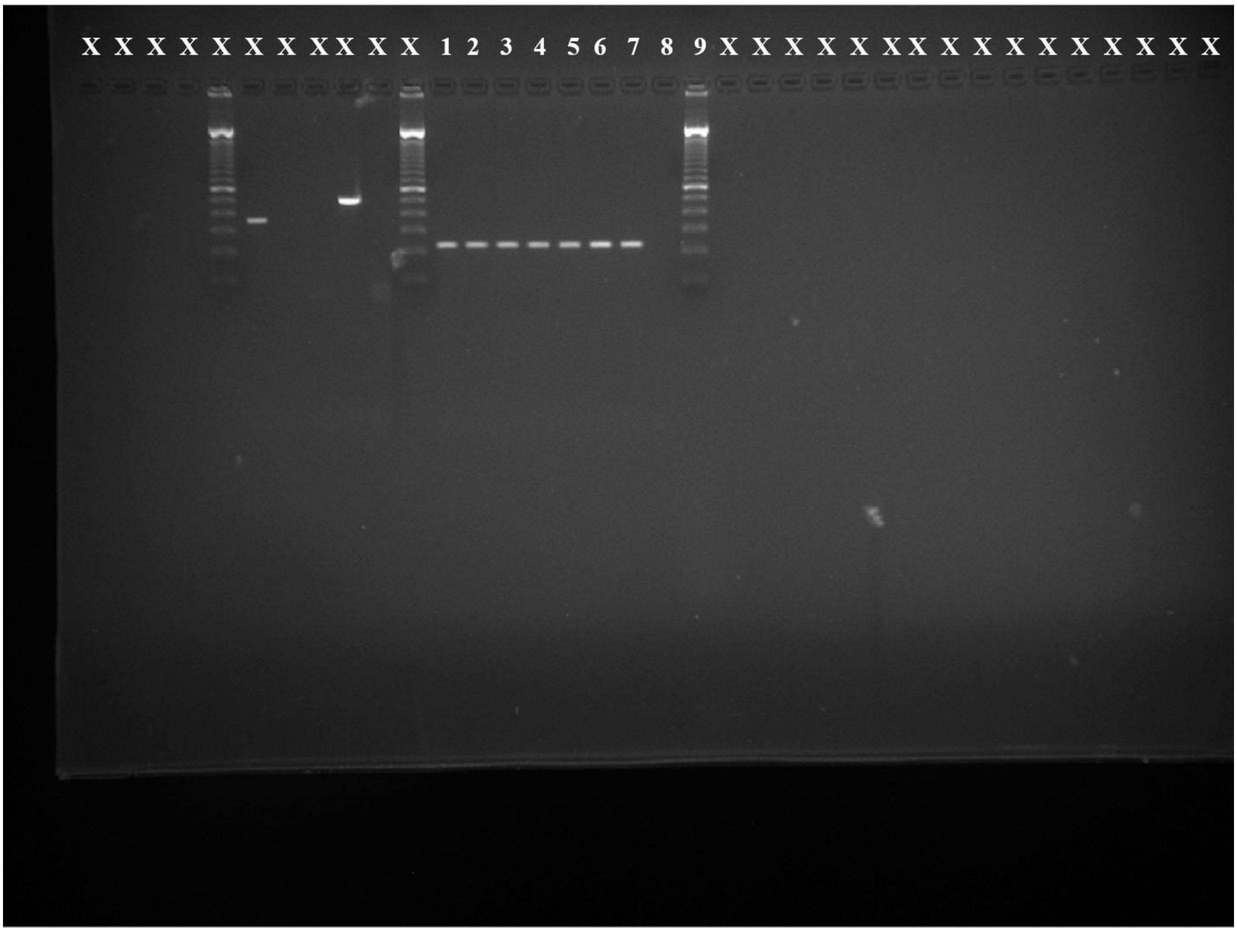

Original gel image supporting Fig 3. Electrophoresis of PCR amplicons on 1.5 % agarose gel stained with ethidium bromide. Gel Doc XR+ System (Bio Rad) with Quantity One 1-D Analysis Software were used to capture the image. Lane 1, *Yersinia pestis* (39/15); Lane 2, *Y. pestis* (59/15); Lane 3, *Y. pestis* (56/15); Lane 4, *Y. pestis* (69/15); Lane 5, *Y. pestis* (70/15); Lane 6, *Y. pestis* (72/15); Lane 7, *Y. pestis* (73/15); Lane 8, no DNA template; Lane 9, 100 bp DNA ladder; 27 Lanes X in order, empty lane, empty lane, empty lane, empty lane, 100 bp DNA ladder, *Escherchia coli*, Extraction control, *Citrobacter diversus*, *Y. pestis* (59/15), no DNA template, 100 bp DNA ladder, empty lane, empty lane, empty lane, empty lane, empty lane, empty lane, empty lane, empty lane, empty lane, empty lane, empty lane, empty lane, empty lane, empty lane, empty lane, empty lane.

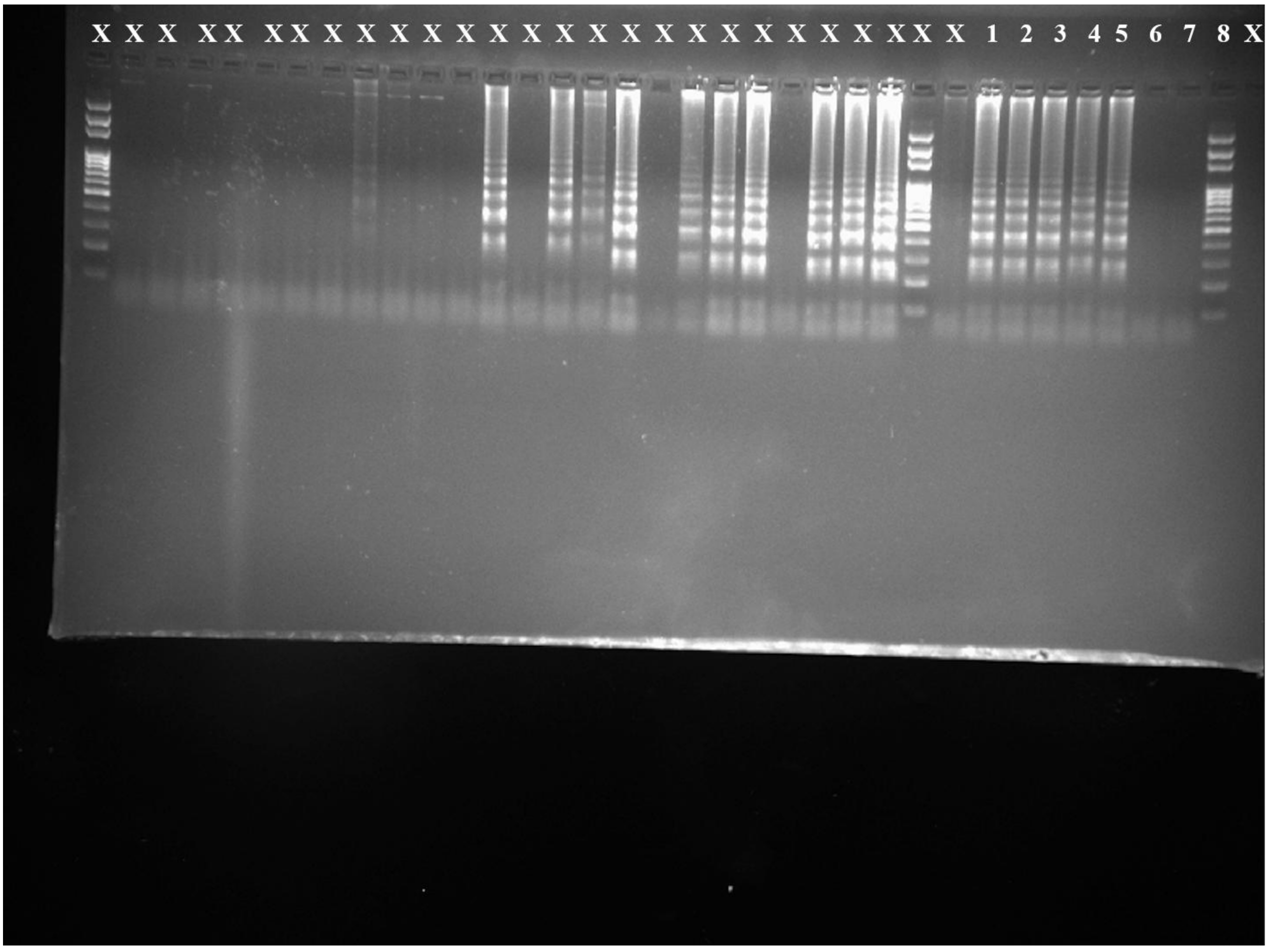

Original gel image supporting Fig 4 (B). Electrophoresis of LAMP amplicons on 1.5 % agarose gel stained with ethidium bromide. Gel Doc XR+ System (Bio Rad) with Quantity One 1-D Analysis Software were used to capture the image. Lane 1, *Yersinia pestis* (59/15) undiluted; Lane 2, *Y. pestis* (59/15) diluted 10^{-1}; Lane 3, *Y. pestis* (59/15) diluted 10^{-2}; Lane 4, *Y. pestis* (59/15) diluted 10^{-3}; Lane 5, *Y. pestis* (59/15) diluted 10^{-4}; Lane 6, *Y. pestis* (59/15) diluted 10^{-5}; Lane 7, no DNA template; Lane 8, 100 bp DNA ladder; 28 Lanes X in order, 100 bp DNA ladder, no DNA template, *Y. pestis* (59/15), *Y. pestis* (59/15), *Y. pestis* (59/15), no DNA template, *Y. pestis* (59/15), *Y. pestis* (59/15), *Y. pestis* (59/15), no DNA template, *Y. pestis* (59/15), *Y. pestis* (59/15), *Y. pestis* (59/15), no DNA template, *Y. pestis* (59/15), *Y. pestis* (59/15), *Y. pestis* (59/15), no DNA template, *Y. pestis* (59/15), *Y. pestis* (59/15), *Y. pestis* (59/15), no DNA template, *Y. pestis* (59/15), *Y. pestis* (59/15), *Y. pestis* (59/15), 100 bp DNA ladder, no DNA template, empty lane.

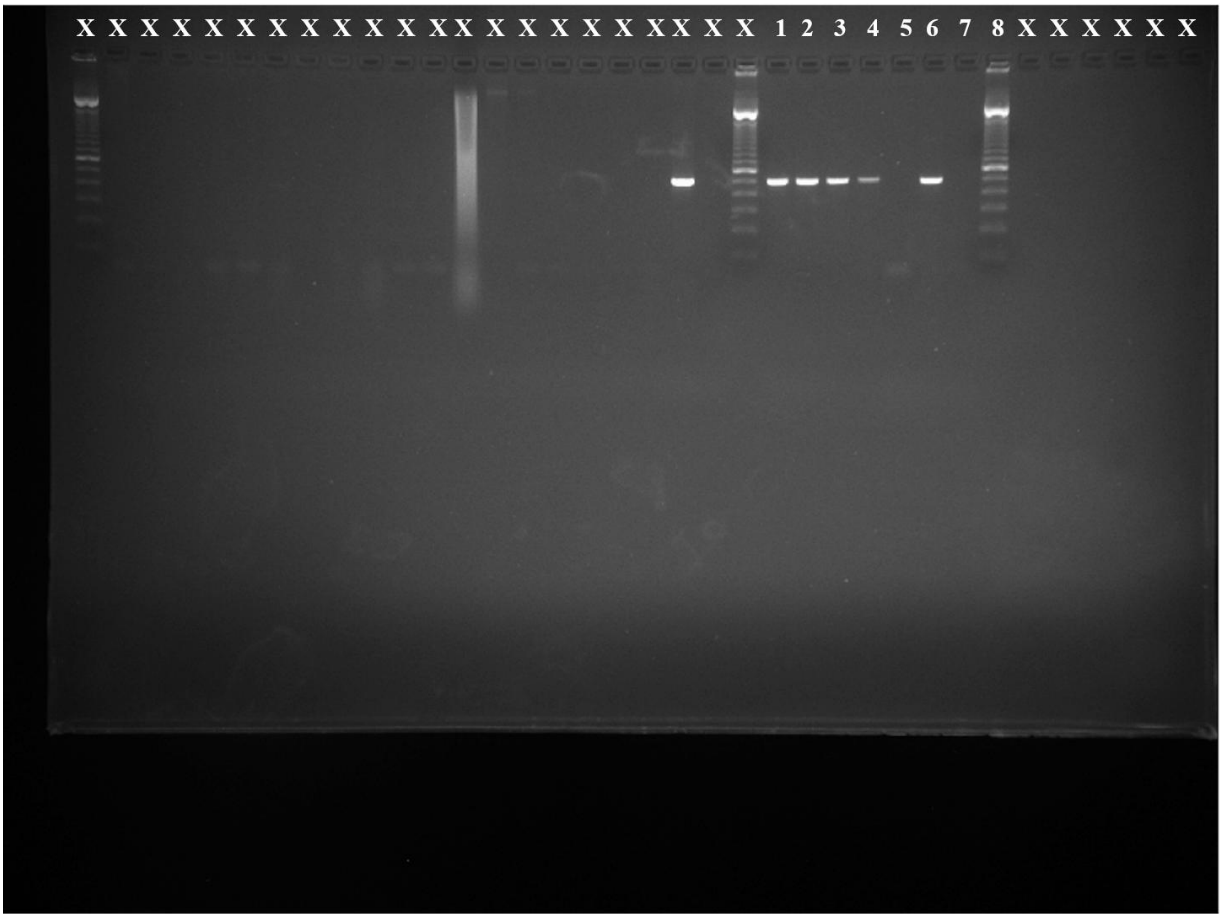

Original gel image supporting Fig 4 (C). Electrophoresis of PCR amplicons on 1.5 % agarose gel stained with ethidium bromide. Gel Doc XR+ System (Bio Rad) with Quantity One 1-D Analysis Software were used to capture the image. Lane 1, Yersinia pestis (59/15) diluted 10^{-1}; Lane 2, Y. pestis (59/15) diluted 10^{-2}; Lane 3, Y. pestis (59/15) diluted 10^{-3}; Lane 4, Y. pestis (59/15) diluted 10^{-4}; Lane 5, Y. pestis (59/15) diluted 10^{-5}; Lane 6, Y. pestis (59/15) non diluted; Lane 7, no DNA template; Lane 8, 100 bp DNA ladder; 28 Lanes X in order, 100 bp DNA ladder, *Yersinia enterocolitica*, extraction control, *Yersinia pseudotuberculosis*, *Enterobacter cloacae*, *Escherichia coli*, *Shigella sonnei, Proteus mirabilis*, *Serratia odorifera*, *Serratia marescens*, *Pseudomonas aeruginosa*, *Staphylococcus aureus*, *Mycobacterium tuberculosis* (1), *M. tuberculosis* (2), *M. tuberculosis* (3), *M. tuberculosis* (4), *Plasmodium vivax*, *Plasmodium falciparum*, *Taenia solium, Y. pestis* (59/15), no DNA template, 100 bp DNA ladder, empty lane, empty lane, empty lane, empty lane, empty lane, empty lane.

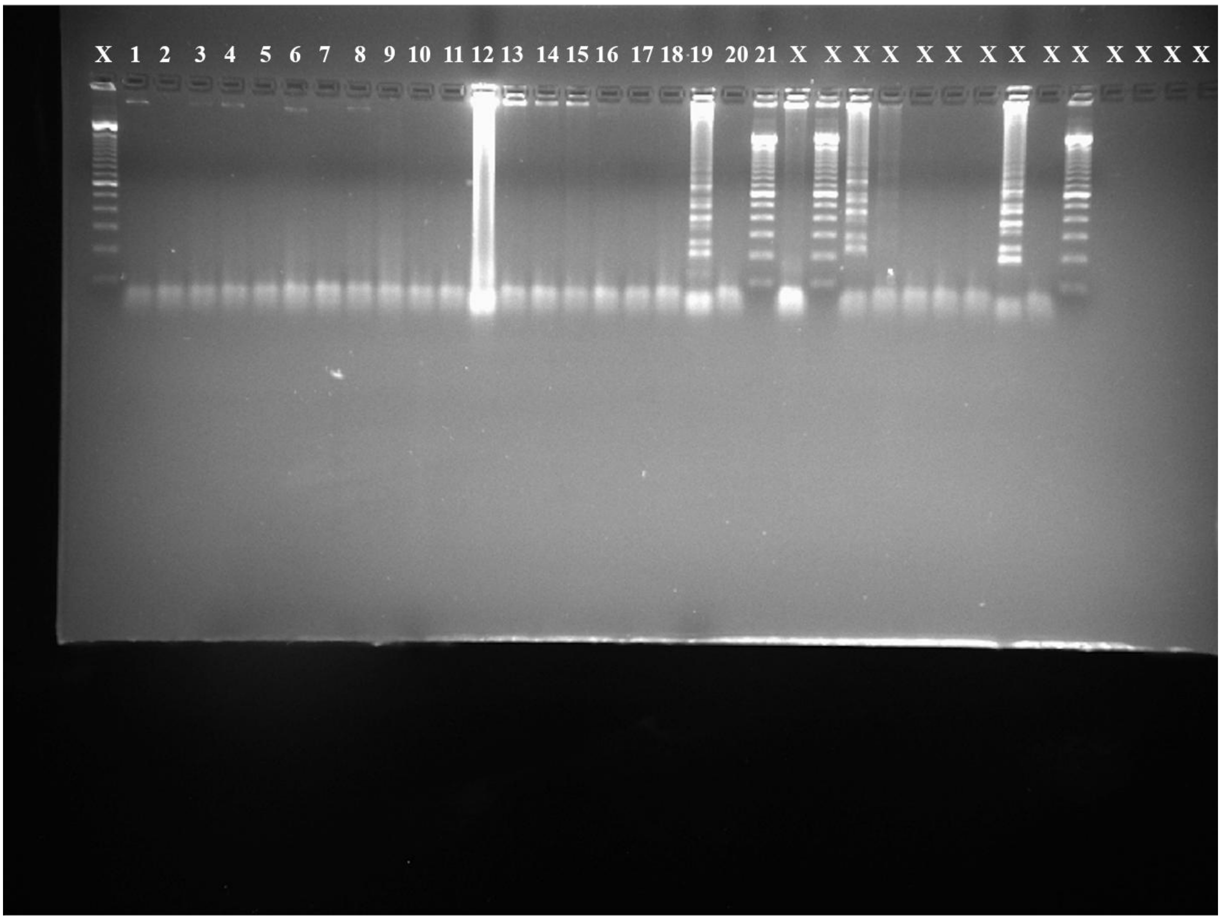

Original gel image supporting Fig 5 (B). Electrophoresis of LAMP amplicons on 1.5 % agarose gel stained with ethidium bromide. Gel Doc XR+ System (Bio Rad) with Quantity One 1-D Analysis Software were used to capture the image. Lane 1, *Yersinia enterocolitica*; Lane 2, extraction control; Lane 3, *Yersinia pseudotuberculosis*; Lane 4, *Enterobacter cloacae*; Lane 5, *Escherichia coli*; Lane 6, *Shigella sonnei*; Lane7, *Proteus mirabilis*; Lane 8, *Serratia odorifera*; Lane 9, *Serratia marescens*; Lane 10, *Pseudomonas aeruginosa*; Lane 11, *Staphylococcus aureus*; Lane 12, *Mycobacterium tuberculosis* (1); Lane 13, *M. tuberculosis* (2); Lane 14, *M. tuberculosis* (3); Lane 15, *M. tuberculosis* (4); Lane 16, *Plasmodium vivax*; Lane 17, *Plasmodium falciparum*; Lane 18, *Taenia solium*; Lane 19, *Y. pestis* (59/15); Lane 20, no DNA template; Lane 21, 100 bp DNA ladder; 15 Lanes X in order, 100 bp DNA ladder, *E. coli* 2nd test, 100 bp DNA ladder, *Y. pestis* (diluted 10^{-1}), *Y. pestis* (diluted 10^{-2}), *Y. pestis* (diluted 10^{-3}), *Y. pestis* (diluted 10^{-4}), *Y. pestis* (diluted 10^{-5}), *Y. pestis* non diluted, No DNA template, 100 bp DNA ladder, empty lane, empty lane, empty lane, empty lane.
