# Supplementary material for: Development and evaluation of loop-mediated isothermal amplification for detection of Yersinia pestis in plague biological samples
Source: PLoS One. 2020 Aug 18;15(8):e0237655. doi: 10.1371/journal.pone.0237655 (PMC7437451; doi:10.1371/journal.pone.0237655)
Supplement: Attachment — Submitted filename: Response to Reviewers.docx [file pone.0237655.s004.docx]

**Response to Reviewers**

PONE-D-20-06493
Development and evaluation of loop-mediated isothermal amplification for detection of Yersinia pestis in plague biological samples
PLOS ONE

**PLoS ONE Academic editor requirements**

In particular you are asked to further study the diagnostic performance of the developed assay by incliuding more samples, in particular of healthy endemic controls and other diseases.

Answer: For ethical reasons, it was not possible to obtain negative control adenitis aspirates from patients without suspected plague. Therefore, samples from negative patients tested on our LAMP caf1 were constituted of samples from patients not suspected for plague which were archived at the Clinical Center of Biology (Institut Pasteur de Madagascar) for other analysis: sputum (n= 18) and pus (n= 29)

We were also able to add 47 patient samples with other diseases to test on our LAMP caf1. They are distributed as follows: schistosomiasis (n= 7), taeniasis (n= 3), cysticercosis (n= 8), filariosis (n= 1) and tuberculosis (n= 28). They were kindly provided by other research units from the Institut Pasteur de Madagascar from their samples collection.

The employed statistics require further attention

Answer: Thank you for pointing this out. Estimations of the Sensibility and specificity of the LAMP caf1 were reviewed using appropriate statistical tests. In this purpose, we replaced in the “Materials and Methods” section the paragraph “Sensitivity and specificity of LAMP caf1” to “Statistical analysis” which highlighted the method used for sensitivity and specificity estimation (see page 11, line 206 of the revised manuscript).

The issues about diagnostic performance should be addressed in an inteligent manner

Answer: Following these requirements from the Academic editor and reviewers, statements on the diagnostic performance were added in the manuscript for better clarification.

All issues and points raised by the reviewers must be addressed

Answer: Thank you very much. We have answered to all the issues and questions raised by the reviewers. See our Answers below.

The figures need to be of better quality

Answer: Thank you. We also reviewed all the figures to meet the journal’s requirements for quality.

**Journal requirements**

When submitting your revision, we need you to address these additional requirements:

1. Please ensure that your manuscript meets PLOS ONE's style requirements, including those for file naming. The PLOS ONE style templates can be found at http://www.plosone.org/attachments/PLOSOne_formatting_sample_main_body.pdf and http://www.plosone.org/attachments/PLOSOne_formatting_sample_title_authors_affiliations.pdf

Answer: The revised manuscript meets PLOS ONE's style requirements

2. PLOS ONE now requires that authors provide the original uncropped and unadjusted images underlying all blot or gel results reported in a submission’s figures or Supporting Information files. This policy and the journal’s other requirements for blot/gel reporting and figure preparation are described in detail at https://journals.plos.org/plosone/s/figures#loc-blot-and-gel-reporting-requirements and https://journals.plos.org/plosone/s/figures#loc-preparing-figures-from-image-files. When you submit your revised manuscript, please ensure that your figures adhere fully to these guidelines and provide the original underlying images for all blot or gel data reported in your submission. See the following link for instructions on providing the original image data: https://journals.plos.org/plosone/s/figures#loc-original-images-for-blots-and-gels.

Answer: Original underlying images for all gel data reported in the revised manuscript are provided as Supporting Information file (pdf file) with specific details.

In your cover letter, please note whether your blot/gel image data are in Supporting Information or posted at a public data repository, provide the repository URL if relevant, and provide specific details as to which raw blot/gel images, if any, are not available. Email us at plosone@plos.org if you have any questions.

Answer: Gel image data in Supporting Information was mentioned in the revised cover letter

3. We note you have two different tables labeled as Table 3. Please ensure that all your tables are numbered separately.

Answer: The numbers of tables were corrected accordingly:

Page 11, line 199 (previous version) and Page 11, line 205 (revised version): Table 3. “Classification of the LAMP results” was deleted.

The paragraph entitled “Sensitivity and specificity of LAMP caf1” has been renamed to “Statistical analysis” to highlight the statistical method used to assess the performance of LAMP *caf1*.

Page 15, line 272 (previous version) and Page 15, line 279 (revised version): Table 3. now refers to “Evaluation of LAMP caf1 performance”

**Review Comments to the Authors**

**Reviewer #1**: The manuscript by Lovasoa Nomena Randriantseheno describes the development of a LAMP assay that would be able to detect Y. pestis in human biological samples, and thereby improving the diagnosis of plague and circumventing cumbersome bacteriological culture.

1- The authors claim that no ethical approval is needed for his study and that there is no informed consent needed from study cases. Although I believe that this might be right in this specific case a document supporting this would be good to have.

Answer: Thank you for asking for this supporting document. Although not available online, the French hard copy version of the Plague National Control Program (Edition 2012) of the Malagasy Ministry of Public Health stated at page 7, paragraph 4.4 that the notification of each plague case is mandatory at national level. It consists in recording all the necessary and useful information concerning each suspect plague patient as well as the epidemiological context in the official individual declaration form, a copy of which will be sent with the sample to the central laboratory for plague for confirmation, the surveillance of the sensitivity of strains to antibiotics and for the international weekly declaration.

We would also want to notice that these statements were already mentioned as such in other publications (see list below) using biological samples and/ or *Y. pestis* strains derived from plague suspected patients of Madagascar, see below:

- Vogler AJ, Andrianaivoarimanana V, Telfer S, Hall CM, Sahl JW, Hepp CM, Centner H, Andersen G, Birdsell DN, Rahalison L, Nottingham R, Keim P, Wagner DM, Rajerison. M. [Temporal phylogeography of Yersinia pestis in Madagascar: Insights into the long-term maintenance of plague.](https://www.ncbi.nlm.nih.gov/pubmed/28873412) PLoS Negl Trop Dis. 2017 Sep 5;11(9):e0005887. doi: 10.1371/journal.pntd.0005887. eCollection 2017 Sep.

- Vogler AJ, Chan F, Wagner DM, Roumagnac P, Lee J, Nera R, Eppinger M, Ravel J, Rahalison L, Rasoamanana BW, Beckstrom-Sternberg SM, Achtman M, Chanteau S, Keim P. [Phylogeography and molecular epidemiology of Yersinia pestis in Madagascar.](https://www.ncbi.nlm.nih.gov/pubmed/21931876) PLoS Negl Trop Dis. 2011 Sep;5(9):e1319. doi: 10.1371/journal.pntd.0001319. Epub 2011 Sep 13.

2- The work aimed to develop a rapid, simple and sensitive/specific LAMP method for the detection of the caf1 gene sequence that is specific to *Y. pestis* and to evaluate its performance on biological samples from plague suspected patients from Madagascar. However, apparently previously a LAMP assay has been developed see line 100. The authors should specify why they undertook the current study. What is their novel approach.

Answer: References cited at line 100-101 are related to the development of *Yersinia pestis* LAMP assay using pure cultures of *Y. pestis* (ref#20: de Lira Nunes M et al., 2014) and simulated samples (ref#21: Feng N et al, 2017) which are already clearly stated at the introduction section. These LAMP assays were not evaluated on biological samples collected from plague suspected patients which was the aim of our study. Therefore we think that our LAMP technique was the first which was evaluated on true plague samples as they are intended to be used later.

3- The development of the LAMP assay is straightforward and well described.

Diagnostic characteristics of the test would have been more convincing if also **patient samples of cases with other diseases were tested**. Can the authors do this?

Answer: Thank you for this interesting suggestion. We were able to add 47 patient samples with other diseases to test on our LAMP caf1. They are distributed as follows: schistosomiasis (n = 7), taeniasis (n = 3), cysticercosis (n = 8), filariosis (n = 1) and tuberculosis (n = 28). They were kindly provided by other research units from the Institut Pasteur de Madagascar from their samples collection.

They did not show cross reactions.

4- Sensitivity and specificity should be presented with 95% confidence intervals (also in the abstract). Agreement between tests (kappa values) must be estimated and presented. In particular there should be a clear definition of the reference test. This is now presented partly in the discussion but must be included in the Methods section and presented in the Results section.

Answer: Thank you for pointing this out. The presentation of Sensitivity and specificity of the test was reviewed according to the requirement of the reviewers i.e. using 95% confidence intervals. Agreement between tests (kappa values) were also estimated and presented in all the section of the manuscript (Abstract, Materials and Methods, Results and Discussion).

We used bacteriology with isolation of *Y. pestis* strain as reference test for plague diagnosis which is the gold standard as per the WHO (Wer, 2006)

5- Figures are not of very good quality. This must be improved.

Answer: We agree with the reviewer’s comment. They were improved to meet the journal requirement accordingly.

**Reviewer #2**: The manuscript addresses the development of a loop-mediated isothermal amplification (LAMP) test to aid in the diagnosis of Plague by detecting Yersinia pestis nucleic acids in clinical samples.
The authors explain the advantages of polymerase chain reaction (PCR) in the diagnosis of Plague, in opposition to culture and antigen detection rapid diagnostic test (RDT). The aim of the study is to develop a LAMP method that being simpler than PCR can replace it and implemented at lower levels of the health system, provided it has good diagnostic performance.
The methodology used is appropriate, but I would like to discuss some aspects of this work:

1) According to the authors PCR alone is not enough to confirm a diagnosis of Plague and this requires also a positive RDT result or a 4X increase in antibody titre, why is this required? Please, explain. Why not aiming at a LAMP method that can be used alone as confirmatory tool?

Answer: We refer to the plague case definition according to the WHO (see WER, 2006) which stated that a confirmed plague case requires: a culture positive with isolation of *Y. pestis* OR a combination of PCR and RDT positive OR a 4X increase in antibody titre. To date, there is no updated guideline from the WHO which can validate the use of PCR alone as a confirmatory test. However, as it was designed to be used at least at the district hospital, at the patient’s bedside, the plague LAMP method can help physicians (working at the health centers in remote plague areas) to confirm plague cases when combined with F1 RDT. Indeed, F1 RDT is already widely used in plague endemic areas since 2002.

2) The LAMP method proposed is home brewed, will this be accepted by the Ministry of Health? Is the PCR method also home brewed?

Answer: We think that this will be accepted by the MoH since almost biological tools used at the CLP for plague diagnosis followed home-made protocols. In addition, they were all validated on biological samples as required by the WHO.

3) When cross-reactions were assessed was DNA preparation and measurement the same for all samples? This is not clear.

Answer: Fourteen pathogens were tested for cross-reactions with LAMP caf1. Most of these pathogens were cultured in CLP and DNA extraction was performed using DNeasy Blood & Tissue Kits. However, for *Taenia solium*, *Plasmodium vivax*, *Plasmodium falciparum* and *Mycobacterium tuberculosis*, DNA extraction was conducted by other research units of the Institut Pasteur de Madagascar following their own protocols. The methods may be slightly different compared to our extraction protocol: DNeasy Blood & Tissue Kit (Qiagen Kit) used for *Taenia solium*, *Plasmodium vivax*, *Plasmodium falciparum* and an in-house protocol for *Mycobacterium tuberculosis*.

However, DNA measurements of all these pathogens were performed using Nanodrop 2000 but no DNA concentration adjustment was done before testing. Therefore, we added clarifications in the manuscript according to the reviewer’s comment.

4) LAMP shows lower sensitivity than culture. Is not this a suboptimal performance?

Answer: It is true that compared to culture, LAMP caf1 shows a lower sensitivity. However, in most of countries affected by plague, having additional test which can be used at the peripheral level allows health care workers to confidently rule in cases mainly for pneumonic plague, a severe and contagious disease, which may be clinically confused with acute respiratory infection (IRA).

Also, the use of SYBRGreen is a big limitation, the authors suggest to quantify DNA first and adjust DNA concentration, which is not ideal for a point-of-care (POC) test. Also, for coloured DNA samples analysis of amplified products by PCR is also suggested, which again is far from a POC.

Answer: We actually highlighted some problems related to the use of Sybr Green I during this study. While testing *M. tuberculosis* DNA for cross-reactions, we found that high concentration of DNA can lead to a false positive result with Sybr Green I. We proposed to quantify the DNA extract before amplification but for point-of-care diagnostic, we understood that this is not the ideal scenario. That is why we proposed an alternative method consisting in adding Sybr Green I to an aliquot of DNA before starting the amplification reaction. This is to make sure that the DNA concentration of the pre-treated sample is not too high and will not lead to a false positive result, if Sybr Green I turns green with this prior test, a simple dilution of the pre-treated sample is needed before starting the amplification. Regarding the dark colored sample, of the 113 samples tested, only one sample was dark colored and led to misinterpretation of the colorimetric result. For this sample, we run LAMP amplicons in a gel electrophoresis and saw the ladder-like pattern to classify it as a positive reaction. This case is an exception. Indeed, the biological samples collected for plague tests are either bubo aspirates or sputum samples which are usually not dark colored. For the case of septicemic plague, which is rarely reported, blood samples are collected and additional steps in the pre-treatment procedure can be applied to avoid such problems as reported previously (Viana GMR, Silva-Flannery L, Lima Barbosa DR, et al. Field evaluation of a real time loop-mediated isothermal amplification assay (RealAmp) for malaria diagnosis in Cruzeiro do Sul, Acre, Brazil. PLoS One. 2018;13(7):e0200492. Published 2018 Jul 11. doi:10.1371/journal.pone.0200492). However, no blood samples were included in our study.

5) Sensitivity and specificity are not estimated with confidence intervals, please apply the statistics needed for this.

Answer: Thank you for this remark. Sensitivity and specificity are now estimated with 95% confidence intervals as recommended by the reviewers. Agreement between tests (kappa values) were also estimated and presented in the whole manuscript.

6) The resulting LAMP test, as it is, does not seem to bring any advantage to the diagnosis of Plague

Answer: We don’t agree with the reviewer’s comment. According to the performance of the LAMP test developped in this study (Sensitivity of 97.9% and Specificity of 94.6% compared to the gold standard which is the bacteriological culture) and its practicability particularly for low resource countries such as Madagascar, it is a promising technology for plague diagnosis. Indeed, low income countries where such infectious disease is still endemic have a urgent need to develop diagnostic tool which can be performed at the level of the district hospital without the use of sophisticated equipment and devices. In addition to the F1RDT (already used at the level of plague endemic districts), the implementation of LAMP PCR will allow the confirmation of plague cases on site. This will significantly reduce the time for confirmation and therefore the implementation of appropriate preventive measures to stop the transmission.
