# Supplementary material for: Development and evaluation of loop-mediated isothermal amplification for detection of Yersinia pestis in plague biological samples
Source: PLoS One. 2020 Aug 18;15(8):e0237655. doi: 10.1371/journal.pone.0237655 (PMC7437451; doi:10.1371/journal.pone.0237655)
Supplement: Attachment — Submitted filename: Response to Reviewers.docx [file pone.0237655.s005.docx]

**Response to Reviewers**

PONE-D-20-06493R1
Development and evaluation of loop-mediated isothermal amplification for detection of Yersinia pestis in plague biological samples
PLOS ONE

**Review Comments to the Author**

**Reviewer #1**: Most of my comments have been addressed and some addiitonal samples seem to be analysed. I would like to see a statement on the patients'consent that these addiitonal sample could have been used for this study (and/or ethical review outcome that supports this).

Answer: Additional samples used for this study have been anonymized and aggregated precluding identification of patients at individual level. They are part of residual samples from:

- Research project authorized for further use in future research (following the reference # 041-MSANP/CERBM du 08 juin 2017 from the Ethics Committee for Biomedical Research of the Malagasy Ministry of Public Health).

- Tuberculosis confirmation: mandatory samples collection as part of Madagascar’s routine surveillance system under national public health law.

They were all provided in the purpose of Public Health improvement in Madagascar

Also the relevant part of the document the authors refer to that cover the ethical approval for the study should be uploaded as suplementary material
Answer: A copy of the Plague National Control Program (Edition 2012) of the Malagasy Ministry of Public Health has been uploaded as supplementary material (**S1 Appendix. Plague National Control Program)**

English may require some attention
Answer: The whole manuscript has now been reviewed by Dr Dawn Birdsell - a native English speaking scientist- for English improvement

Quality of figures remains a concenr
Answer: All figure files have been greatly improved in terms of resolution to meet the Journal requirements. They have also been uploaded to the Preflight Analysis and Conversion Engine (PACE) digital diagnostic tool, <https://pacev2.apexcovantage.com/> as required by the Journal
